# Performance characteristics of the first Food and Drug Administration (FDA)-cleared digital droplet PCR (ddPCR) assay for *BCR*::*ABL1* monitoring in chronic myelogenous leukemia

**DOI:** 10.1371/journal.pone.0265278

**Published:** 2022-03-17

**Authors:** Dawne N. Shelton, Prasanthi Bhagavatula, Nathan Sepulveda, Lan Beppu, Shital Gandhi, Dahui Qin, Scott Hauenstein, Jerald Radich

**Affiliations:** 1 Digital Biology Group, Bio-Rad Laboratories, Pleasanton, California, United States of America; 2 Clinical Research Division, Fred Hutchinson Cancer Research Center, Seattle, Washington, United States of America; 3 Department of Pathology, Moffitt Cancer Center, Tampa, Florida, United States of America; Istanbul University-Cerrahpaşa, Cerrahpaşa Faculty of Medicine, TURKEY

## Abstract

Chronic myelogenous leukemia (CML) is a hematopoietic stem cell malignancy that accounts for 15–20% of all cases of leukemia. CML is caused by a translocation between chromosomes 9 and 22 which creates an abnormal fusion gene, *BCR*::*ABL1*. The amount of *BCR*::*ABL1* transcript RNA is a marker of disease progression and the effectiveness of tyrosine kinase inhibitor (TKI) treatment. This study determined the analytical and clinical performance of a droplet digital PCR based assay (QXDx BCR-ABL %IS Kit; Bio-Rad) for *BCR*::*ABL1* quantification. The test has a limit of detection of MR4.7 (0.002%) and a linear range of MR0.3–4.7 (50–0.002%IS). Reproducibility of results across multiple sites, days, instruments, and users was evaluated using panels made from *BCR*::*ABL1* positive patient samples. Clinical performance of the assay was evaluated on patient samples and compared to an existing FDA-cleared test. The reproducibility study noted negligible contributions to variance from site, instrument, day, and user for samples spanning from MR 0.7–4.2. The assay demonstrated excellent clinical correlation with the comparator test using a Deming regression with a Pearson R of 0.99, slope of 1.037 and intercept of 0.1084. This data establishes that the QXDx^™^ BCR-ABL %IS Kit is an accurate, precise, and sensitive system for the diagnosis and monitoring of CML.

## Introduction

Chronic myeloid leukemia is a myeloproliferative stem cell disorder characterized by the t(9;22) reciprocal translocation. On a molecular basis, this produces a novel *BCR*::*ABL1* tyrosine kinase fusion gene which causes an expansion in the white blood cell compartment through constitutive activation of the *ABL1* kinase. Without definitive therapy, most cases of CML will progress to acute leukemia. The advent of tyrosine kinase inhibitor (TKI) therapy has revolutionized the treatment of CML, allowing most patients to enjoy a near normal life expectancy. The successful management of CML relies on regular and precise monitoring of the *BCR*::*ABL1* mRNA in the peripheral blood. The National Comprehensive Cancer Network (NCCN) and European Leukemia Network (ELN) have clear guidelines for initial therapy, monitoring intervals, changing therapy due to poor response or potential discontinuation for cases with a deep molecular response [1, 2].

In greater than 95% of cases, *BCR*::*ABL1* transcripts arise from the fusion of exons 13 or 14 of the *BCR* gene to exon 2 of the *ABL1* gene (e13a2 and e14a2, respectively), thus coding for the 210-kDa protein product [3]. In Ph-positive acute lymphoblastic leukemia, the major isoform results from the fusion of exon 1 of the *BCR* gene with exon 2 of the *ABL1* gene (e1a2), yielding a 190-kDa protein product [3, 4]. Quantitative reverse transcriptase PCR (RT-qPCR) of *BCR*::*ABL1* mRNA is a sensitive method for monitoring CML, yet RT-qPCR technical assay variation from lab to lab yielded log fold differences, leading to data that was difficult to interpret from lab to lab [5, 6]. To address these problems, a large international team of CML researchers developed and validated the International Standard scale for *BCR*::*ABL1* to minimize this variation [7].

When optimized properly, the RT-qPCR assay can detect the *BCR*::*ABL1* transcript in a background of ~100,000 total mRNA transcripts [8]. CML disease burden is monitored by the reduction in *BCR*::*ABL1* transcripts from a standardized baseline value (the International Scale) as “IS%”. The amount of disease burden reduction is denoted by MR (molecular response) followed by the log amount of reduction. For example, while not used as a clinical endpoint, MR2 would refer to a two-log reduction of *BCR*::*ABL1/BCR* from 100%IS to ≤ 1%IS (MR0 to MR2). The concept of a major molecular response (MMR) was initially defined as a ≥ 3-log reduction in *BCR*::*ABL1*/*BCR* in the IRIS trial and became an established response metric because achievement of MMR correlates favorably with long-term outcomes [9]. This three-log reduction is when *BCR*::*ABL1/BCR* decreases from 100%IS to ≤ 0.1%IS (MR0 to MR3) [10]. Early studies with RT-qPCR examining deep molecular response (DMR) and treatment free remission (TFR) indicated that below MR3, the prognostic value of RT-qPCR is limited, with ~50% of patients responding well to stoppage of therapy [11–14]. Though sensitivity cutoffs for prognostic studies have been at MR4 and MR4.5, there are no statistical differences in success rates between detectable and undetectable *BCR*::*ABL1* using qPCR [15]. Several recent studies with digital PCR indicate a detection and discrimination benefit in detecting MR4 and MR4.5–5 samples (stable DMR), and higher probability to maintain the TFR (below cutoff: 54–86% vs above cutoff: 32–52% treatment) [14, 16–19].

The concept of signal to noise explains the difficulty of detecting rare targets (the signal) in a vast background of non-target (the noise). “Digital” PCR (dPCR) is a methodological approach to improve the signal/noise limitation [20]. The concept is to divide the sample into an excess of partitions, so that each partition houses only a few copies of either the target (signal), or the background (noise). Negative droplets would still contain sample nucleic acids, just not the target amplicons. Amplification yields a positive signal with target amplification (digital code = 1), and no amplification in partitions without the target (0). The Poisson distribution is then used to estimate the initial input target *BCR*::*ABL1* copy numbers. The method has the additional advantages of having low technical variation and calibration curves are not required to yield an absolute numerical value [21, 22]. Technical variation is reduced to near the theoretical minimum for all molecular counting methods, known as subsampling error. This error is dependent on the number of copies in the original sample and follows the formula $CV=\;\frac{1}{\sqrt{copies}}$. Variation in dPCR follows this formula throughout the dynamic range of 1–100,000 copies, falling below 10% CV above 100 copies measured [20–23].

Here we describe an accurate, highly precise, and sensitive FDA-cleared droplet digital PCR (ddPCR) assay for *BCR*::*ABL1*, with a comparison to an FDA-cleared RT-qPCR based assay.

## Materials and methods

### Samples

The method comparison study was conducted between November 2017 and April 2018 at the Fred Hutchinson Cancer Research Center, Seattle, Washington, after obtaining IRB exemption by the Institutional Review Board of Fred Hutchinson Cancer Research Center. In addition, reproducibility testing was performed at the Moffitt Cancer Center, Tampa, Florida and at Bio-Rad Laboratories, Pleasanton, California. All analytical performance studies were performed at Bio-Rad Laboratories, Pleasanton, California. The method comparison samples were a mixture of retrospective and prospectively collected samples from multiple geographic locations in the United States. For prospective collection, venous whole blood (≥3 mL, EDTA) was collected from subjects previously diagnosed as t (9;22) positive CML p210. Additional patient samples were acquired from commercial vendors (negatives from Biological Specialty Corporation and positives from Blood Center Wisconsin) for the construction of contrived sample materials (described in individual sections below) utilized in the analytical studies, as well as the clinical method comparison and reproducibility studies. *BCR*::*ABL1* mRNA was extracted using commercially available RNA extraction reagents: TRIzol Reagent (Thermo Fisher Scientific Inc, Waltham, MA, USA), Promega Maxwell CSC RNA Blood Kit (Promega Inc., Madison, WI, USA), QIAamp RNA Blood Mini Kit (Qiagen Inc., Hilden, Germany) following the manufacturers’ instructions, and the RNA samples were stored at -80°C until use.

### *BCR*::*ABL1* assays

The QXDx BCR-ABL %IS Kit (ddPCR; Bio-Rad Laboratories Inc., Pleasanton, California) (described in more detail below) and the Asuragen QuantideX qPCR BCR-ABL IS kit (RT-PCR, Asuragen Inc., Austin, TX) were performed following the manufacturers’ instructions for the comparison of the clinical samples [24]. Both multiplex assays automatically report their results in MR and %IS according to their respective correction factors (as declared by the manufacturer).

Briefly, for ddPCR, a QXDx AutoDG automated droplet generator (Bio-Rad), C1000 thermal cycler (Bio-Rad), and QXDx Droplet Reader (Bio-Rad) were used; the raw data were analyzed and interpreted using the QXDX Acquisition and Analysis Software v 1.0 (Bio-Rad). Each ddPCR kit contains enough reagents to analyze a total of 84 patient samples on two plates, along with controls (NTC, neg, low positive, high positive) and %IS calibrators (~10%IS, ~0.1% IS). Frozen RNA specimens were adjusted to ~100 ng/uL and then ~1ug RNA was reverse transcribed using QXDx iScript Advanced Reverse Transcriptase (Bio-Rad). After reverse transcription, 16ul of cDNA was transferred to a ddPCR plate containing the ddPCR Master Mix. Samples are then split into two wells per sample (data to be merged during analysis) and the plate sealed for droplet generation. After droplet generation, ddPCR amplification was performed using the *BCR*::*ABL1* ddPCR Amplification Thermal Cycling Protocol for ~2 hours. After end point amplification, the plate was transferred to the QXDx Droplet Reader and the droplets read and analyzed. The QXDx Software performs well, sample, and run quality checks. If both sample wells meet the quality metrics, the data is merged. The software then calculates the *BCR*::*ABL1* and ABL copies, %IS, MR values, and checks the values for acceptable limits for controls and %IS calibrator checks [10]. If these meet the acceptance criteria, the sample results are reported.

The RT-PCR assay was performed on 7500 Fast Dx Real-Time PCR instruments (Applied Biosystems by Thermo Fisher Scientific) and run in standard mode. The manufacturer claims that the assay has limit of detection of MR4.7, a limit of quantitation of MR4.7 and a linear range from MR0.3 to MR4.7 [24].

### Specificity/LOB

Thirty-six CML & *BCR*::*ABL1* negative whole blood samples were extracted by the Maxwell CSC RNA Blood Kit to prepare independent RNA samples which were normalized to 100 ng/uL and tested in duplicate. Water from the ddPCR Kit was used as no-template control (NTC) to prepare 48 samples. Samples were tested by one operator on one ddPCR system with two independent kit lots for a total of 144 tests of the patient samples and 96 tests of the NTC. The analysis is based upon the non-parametric option defined in EP17-A2 [25]. All results (N) were rank ordered and the limit of blank calculated as the sample rank position corresponding to the 0.95*N + 0.5th position corresponding to the desired α = 0:05 (Type I Error) risk probability.

In addition, to determine if the minor e1a2 (p190) and micro e19a2 (p230) variants cross react with the ddPCR Kit, two samples were prepared by blending *in-vitro* transcribed p190 or p230 with RNA extracted from normal, healthy, human blood from two donors. Both the p190 and p230 RNA were *in-vitro* transcribed from plasmids in-house. The p230 (e19a2) variant included the e13 and e14 exons of the *BCR* gene. The concentration of the p190 or p230 variants was tested with variant-specific assays prepared in-house. Four dilutions of each sample were prepared by diluting mutant RNA into negative patient RNA and targeting %*BCR*::*ABL1*/*ABL1* from 0.005–30%. Percent specificity was calculated by subtracting mean variant specific assay ratio from ddPCR and dividing the result by ddPCR.

### Analytical sensitivity

The limit of detection (LOD) was first determined by probit regression analysis and then verified by a nonparametric analysis. For the probit regression analysis, three positive *BCR*::*ABL1* RNA patient sample pools were prepared to achieve MR values at approximately MR4. Pool 1 used a mix of fifteen patients positive for the e13a2 and/or e14a2 variants. Pool 2 contained five patients positive for the e13a2 variant and Pool 3 contained five patients positive for the e14a2 variant. One negative *BCR*::*ABL1* RNA sample pool was prepared and used in the dilution of the positive patient sample pools. Each positive patient *BCR*::*ABL1* RNA pool was serially diluted 1:1 with the negative patient RNA pool. All RNA samples were extracted from whole blood by the Maxwell CSC RNA Blood Kit. Samples were tested in replicates of 20 by one operator on one ddPCR system per sample (three instruments) with four independent kit lots (n = 1920). For each dilution, the number of replicates with and without *BCR*::*ABL1* copies detected was determined. A generalized linear model was used to model percent detection, using the probit link function from the binomial family. The LOD was calculated by interpolating the IS ratio that corresponds to a 95% detection rate.

The LOD was verified by analyzing two patient sample pools representing the e13a2 and e14a2 variants. Each pool consisted of 5 patient RNA samples positive for each variant and diluted in a negative pool of 9 CML-negative patient RNA samples. Sample pools were tested across 2 lots, 1 operator, 1 instrument and 8 calendar days in replicates of 20 per day per sample for a total of 320 tests. All RNA samples were extracted from whole blood by the Maxwell CSC RNA Blood Kit. The study followed CLSI EP17-A2, Evaluation of Detection Capability for Clinical Laboratory Measurement Procedures by Classic approach and Nonparametric analysis to determine LOD, with type 2 error at 5% (25). The LOD is defined as the worst median value of the tested %IS values across each sample pool and each lot.

The limit of quantitation (LOQ) was determined in a study that followed CLSI EP17-A2. The LOQ was determined from a set of dilution series. For each dilution series, up to 15 *BCR*::*ABL1* positive patient RNA samples were mixed and diluted into a pool of 18 *BCR*::*ABL1* negative RNA donor samples as described above. The undiluted *BCR*::*ABL1* positive sample pool was used to determine the starting point of each dilution series, and the expected IS value for each dilution sample was determined from the IS value of the undiluted sample and the dilution factor. Each dilution series contained eight levels. Each sample was tested with four kit lots in at least 20 total replicates. The LOQ is the lowest %IS ratio sample that can be measured with an uncertainty of less than 76%CV (10). With an LoB of zero copies, single molecule detection was anticipated. A single copy would yield a %CV = (√1 / 1) *100, or 100%. With a single copy the %CV is a 100% and corresponds to standard deviation of MR values of ~0.25. With an SD of 0.25, the 95% confidence limit falls within the 0.5 log acceptable clinical difference [24, 26–29]. We set our variation specification to a slightly more conservative 76%.

### Analytical precision and accuracy

Within run (intra-assay), between run (inter-assay), between day (inter-assay), between lot, and total precision was evaluated using positive *BCR*::*ABL1* patient RNA in concentrations that spanned the assay range. Sixteen *BCR*::*ABL1* negative and 100 *BCR-*::*ABL1* positive RNA samples representing both the e13a2 and e14a2 variants were utilized to make pools. All RNA samples were extracted from whole blood by the Maxwell CSC RNA Blood Kit. The positive RNA samples were pooled to create six positive patient RNA pools (MR1-4). Each sample, including control samples, was tested by 2 operators on 2 instruments and with 3 lots of reagents. All samples were tested in triplicate for 3 non-consecutive days. The mean, standard deviation (SD) and coefficient of variation (%CV) were calculated for each sample according to CLSI EP5-A2 prescribed methods for data analysis (3 replicates x 2 operators x 3 days x 3 lots x 2 instruments = 108 data points per sample) [30].

To assess accuracy of copies, a set of five double stranded plasmid DNA standards from European Reference Material (ERM-AD623) was tested on ddPCR. Each sample (n = 5; 10, 100, 1,000, 10,000, 100,000 copies) was tested in replicates of 8 by a single operator on three consecutive days with three different lots of assay reagents (24 measurements per level per lot). The measured and certified value of the concentration of *BCR*::*ABL1* b3a2 transcript of the plasmids are compared to assess the accuracy of ddPCR.

### Linearity

The assay linearity was determined by measuring the %IS of two dilution series, one each for the e13a2 and e14a2 *BCR*::*ABL1* variants. This study followed CSLI EP6-A2 [31]. Each dilution series was prepared by serial dilution of a pool of RNA extracted from five CML-positive human blood samples (target concentration of 50% IS) into a pool of RNA extracted from fifteen CML-negative human blood samples. All RNA samples were extracted from whole blood by the Maxwell CSC RNA Blood Kit. Each positive patient pool was serially diluted 1:2.5 with the negative patient RNA pool and all samples were tested in replicates of four.

### Accuracy and traceability to WHO-IS

ddPCR quantifies *BCR*::*ABL1* on the International Scale and is calibrated to the first World Health Organization (WHO) International Genetic Reference Panel for Quantitation of *BCR*::*ABL1* Total RNA (NIBSC code: 09/130) (https://www.nibsc.org/documents/ifu/09-138.pdf). WHO-IS CF values were initially determined per suggested method for calibration of secondary standards, NIBSC Code: 09/138, Version 4, Dated 13/12/2012; WHO International Standard 1^st^ WHO International Genetic Reference Panel for Quantification of *BCR*::*ABL1* translocation by RT-qPCR [32]. In brief, 5 panels of primary standards were extracted by the Qiagen Rneasy mini kit on 5 different days, converted to 2 batches of cDNA on different days, then tested in duplicate over the course of 10 days. A CF per calibrator secondary lot was generated, and then used to generate manufactured calibrator and control lots and assign %IS values. The manufactured calibrator and controls are then included in the kit lots with a value card that is inputted into the software to apply appropriate limits and CF correction. During development, 7 kit lots were tested with WHO-IS primaries to verify alignment. Each level was tested in four replicates. Regression analysis was performed on the measured values of WHO-IS primary samples against published WHO values and results were compared to the established allowable range. Mean and SD for MR and %IS data was calculated for each of the seven lots. Yearly, lots are verified against the WHO-IS primaries to continue traceability and check alignment to the primary values.

### RNA isolation method comparison

Positive CML blood samples arrived untested within 24 hours of draw with the starting MR level unknown. Three dilutions were created to capture the assay range, targeting above and below the clinical decision point of MR3. One *BCR*::*ABL1* positive whole blood CML patient sample was mixed with one *BCR*::*ABL1* negative whole blood patient sample and diluted to create pools at ~2, 3, and 4 MR. Each test sample was divided into 3 aliquots and assigned to one of three extraction methods: Promega Maxwell^®^ CSC RNA Blood Kit, ThermoFisher TRIzol^™^ Reagent, Qiagen QIAamp RNA Blood Mini Kit. Aliquots were used for several independent extraction events, the number of which varied by dilution level (MR2-4, N = 2, 3, 3 respectively). For each extraction, RNA levels were measured with absorbance at 260nm. Each RNA sample was normalized to 100ng/uL with TE. All purified RNA samples were tested by one operator on one system.

### Multi-site reproducibility

The reproducibility of the ddPCR assay was assessed using a panel of 12 contrived patient samples and four controls. Each contrived sample was prepared by mixing an independent pool of RNA isolated from *BCR*::*ABL1* negative whole blood specimens with an independent pool of RNA isolated from *BCR*::*ABL1* positive whole blood specimens, representing both e13a2 and e14a2 variants. RNA samples from a total of 46 unique *BCR*::*ABL1* negative patients and 112 unique *BCR*::*ABL1* positive patients were used in the study. All RNA samples were extracted from whole blood by the Maxwell CSC RNA Blood Kit. The 12 *BCR*::*ABL1* negative RNA pools each contained between two and six unique RNA specimens, and the 12 *BCR*::*ABL1* positive RNA pools each contained between two and 22 unique RNA specimens. The positive and negative pools were mixed to yield %IS values between approximately 0.004%IS (MR4.4) and 18%IS (MR0.7). The control samples were prepared by mixing RNA isolated from a BCR-ABL negative cell line with RNA from two *BCR*::*ABL1* positive cell lines (e13a2 and e14a2).

Each sample was tested by ddPCR with 2 replicates per run and 2 runs per day for 3 non-consecutive days at 3 sites (one instrument at each site), with one reagent lot for a total 36 replicate tests of each sample and 576 total measurements. Each run was performed by an independent operator (2 operators per site). Repeatability, precision, and reproducibility were analyzed for each sample using the reported MR (observed value) as the outcome variable for the primary analysis.

### Clinical sample method comparison

A total of 155 specimens were collected and RNA extracted using either the Maxwell CSC RNA Blood kit or Trizol, followed by testing on site (96 provided by the sponsor, 59 collected at the site). 139 had evaluable quantitative results on both ddPCR and RT-PCR (3 excluded due to protocol deviations and 13 excluded due non-evaluable results in one or both assays). Results were non-evaluable when *BCR*::*ABL1* was not detected or below the limit of quantitation, or when ABL was not detected or too low. Previously extracted RNA from the samples had been subjected to the same storage and thawing conditions and was run on both ddPCR and RT-PCR. The correlation between quantitative results was evaluated by using Weighted Deming linear regression analysis, and Bland–Altman bias analysis to compare MR values per CLSI- EP9 [33, 34].

In addition, the WHO-IS Genetic Reference Panel for Quantitation of *BCR*::*ABL1* Total RNA (NIBSC code: 09/130) gold standard samples were run at four different MR levels and run in replicates of four (4) on both the predicate RT-PCR and ddPCR systems and examined for bias differences against the assigned values.

## Results

### Analytical specificity

For CML negative samples, out of 144 tests, 141 had no detectable *BCR*::*-ABL1* values (97.9%). Three tests (2.0%), all from one sample, had measurements below the LOD of the test and were reported as “Detected below LLOQ”. All but one NTC sample replicate resulted in zero *BCR*::*ABL1* copies (95/96) and was an obvious spurious droplet in the diagonal. All in-kit calibrator checks and control results met the run acceptance criteria. The numbers of *BCR*::*ABL1* copies detected in the negative sample measurements were ranked and the limit of blank was calculated as the % *BCR*::*ABL1* of the 95^th^ percentile sample, per CLSI. The limit of blank was determined to be zero *BCR*::*ABL1* copies. To ensure ddPCR is specific for the e13a2 and e14a2 major fusion transcripts, experiments were conducted to determine if either the e1a2 or e19a2 variants could be detected by the assay. The measured value in all samples was 0.000% demonstrating the kit detects neither the minor e1a2 (p190) nor micro e19a2 (p230) variants [S1 Table].

### Analytical sensitivity (LOD and LLOQ)

**Fig 1:**
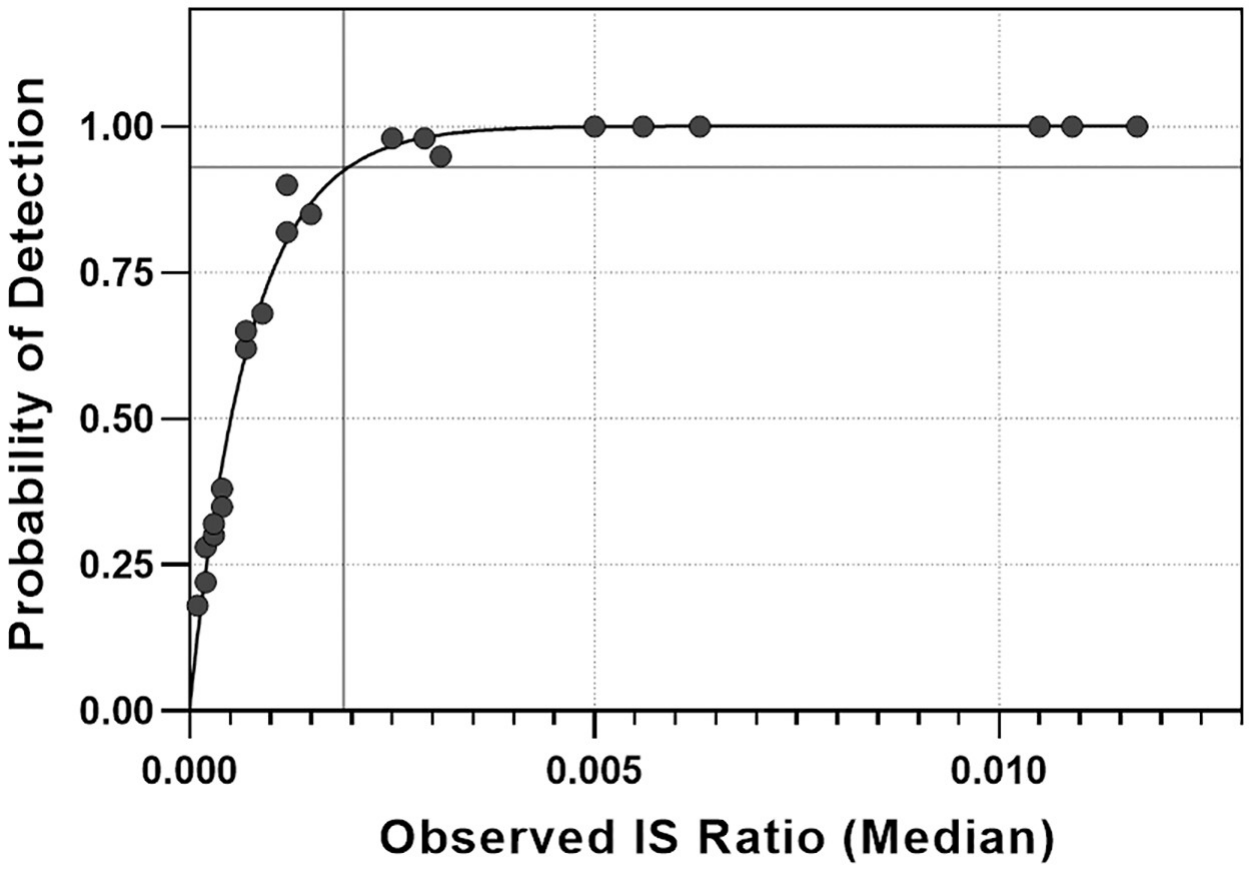
LOD by Probit. A Probit regression analysis was performed using a generalized linear model using a binomial family (solid line through the filled circles that are the median value for the 20 individual replicates). The LOD was calculated by interpolating the IS ratio that corresponds to a 95% detection rate shown as the thin, solid black horizontal and vertical lines.

The initial probit regression model determined the 95% LOD to be 0.0019% IS *BCR*::*ABL1* or MR 4.73 over 4 lots and n = 1920 tests (Fig 1). Verification of this analysis by nonparametric methodology utilizing the combined data from 320 replicates (160 from both e13a2 and e14a2 variants) yielded an LOD for each transcript of 0.002%IS (95% CI 0.0019%-0.0023%) in linear space, MR4.7 in log space, and exactly 3 copies of *BCR*::*ABL1*. For the e13a2 sample, 98.1% (157/160) of tests were above the LOB, with a median MR level of 4.7. For the e14a2 sample, 99.4% (159/160) of tests were above the LOB, with a median MR level of 4.7 (Table 1). In addition to the LOD analysis, the LLOQ was tested by calculating %CV for each variant. The %CV values were sorted by target %IS ratio and grouped by variant or variant and lot. The LLOQ was set to 0.003%IS, corresponding to MR 4.56, as it was the sample with the lowest %IS ratio with a CV less than the target specification of 76% CV [S2 Table].

**Table 1:** LOD verification result summary.

Sample	Valid Measurements	Positive Measurements	Detected %	Median MR Level	Median % IS Ratio
**e13a2**	160	157	98.1%	4.7	0.0018
**e14a2**	160	159	99.4%	4.7	0.0020
**Overall**	320	316	98.8%	4.7	0.0019

### Precision and accuracy

Total MR and %IS precision were calculated for patient samples, cell line control samples and in-kit calibrators/controls. The data for the patient samples is shown in Table 2. Additional precision metrics for MR, including within-run, between run, between operator, between lot and between instruments are displayed for patient and cell line control samples in [S3 Table]. ddPCR demonstrated acceptable precision for all test samples. Within-run variance was the largest contributor to the total precision metric and followed a Poisson distribution; kit lot, operator, instrument, and day having negligible impact on the variability of test samples. The maximum %CV for total precision between MR 0.3-MR4 (actual MR0.7-MR4.1) was 3.95%. Mean MR values were within 0.5 log of the target concentration for all samples, ranging from 0.47 MR (MR 2) to 0.05 (MR 4.7). Variation due to different extraction methods was not included in the total precision study.

**Table 2:** Single-site precision.

Target MR	Mean MR	SD	% CV	Target %IS	Mean %IS	% CV	*N*
**1**	1.4	0.03	2.11	10	3.9880	6.79	100
**2**	2.47	0.05	1.85	1	0.3412	10.40	100
**2.5**	2.80	0.05	1.71	0.32	0.1591	10.83	100
**3**	3.31	0.08	2.42	0.1	0.0501	17.85	100
**3.5**	3.63	0.10	2.83	0.032	0.0242	22.51	100
**4**	4.13	0.16	3.95	0.01	0.0079	36.24	99

**Fig 2:**
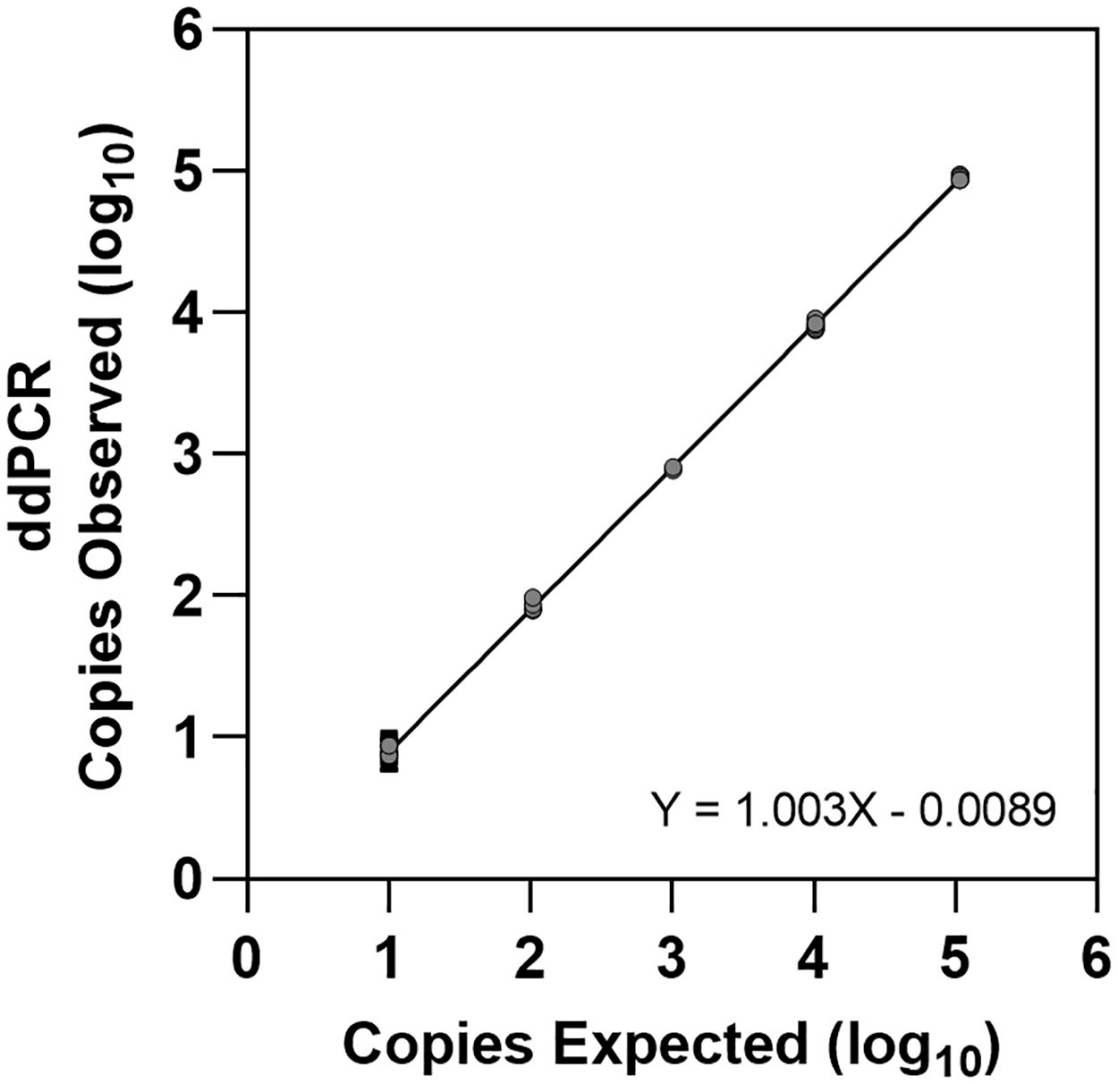
Accuracy of ERM control material. Each sample (10, 100, 1,000, 10,000, 100,000 copies) was tested in replicates of 8 shown with solid grey circles. The solid black line is the linear regression and has a y intercept equal to -0.0089, slope of 1.003 and a R^2^ of 0.9988.

An additional examination of ddPCR accuracy was performed using European Reference Material (ERM-AD623) containing plasmid DNA in concentrations ranging from 10–100,000 copies of *BCR*::*ABL1* (Fig 2). Regression analysis demonstrated a y-intercept of -0.0089 with a slope equal to 1.003; with a R^2^ = 0.9988. The variation across the replicates, as reflected by % CV, ranged from a high of 12.2% at 10 copies to <1% at higher copy numbers [S4 Table].

### Linearity for fusion transcripts

**Fig 3:**
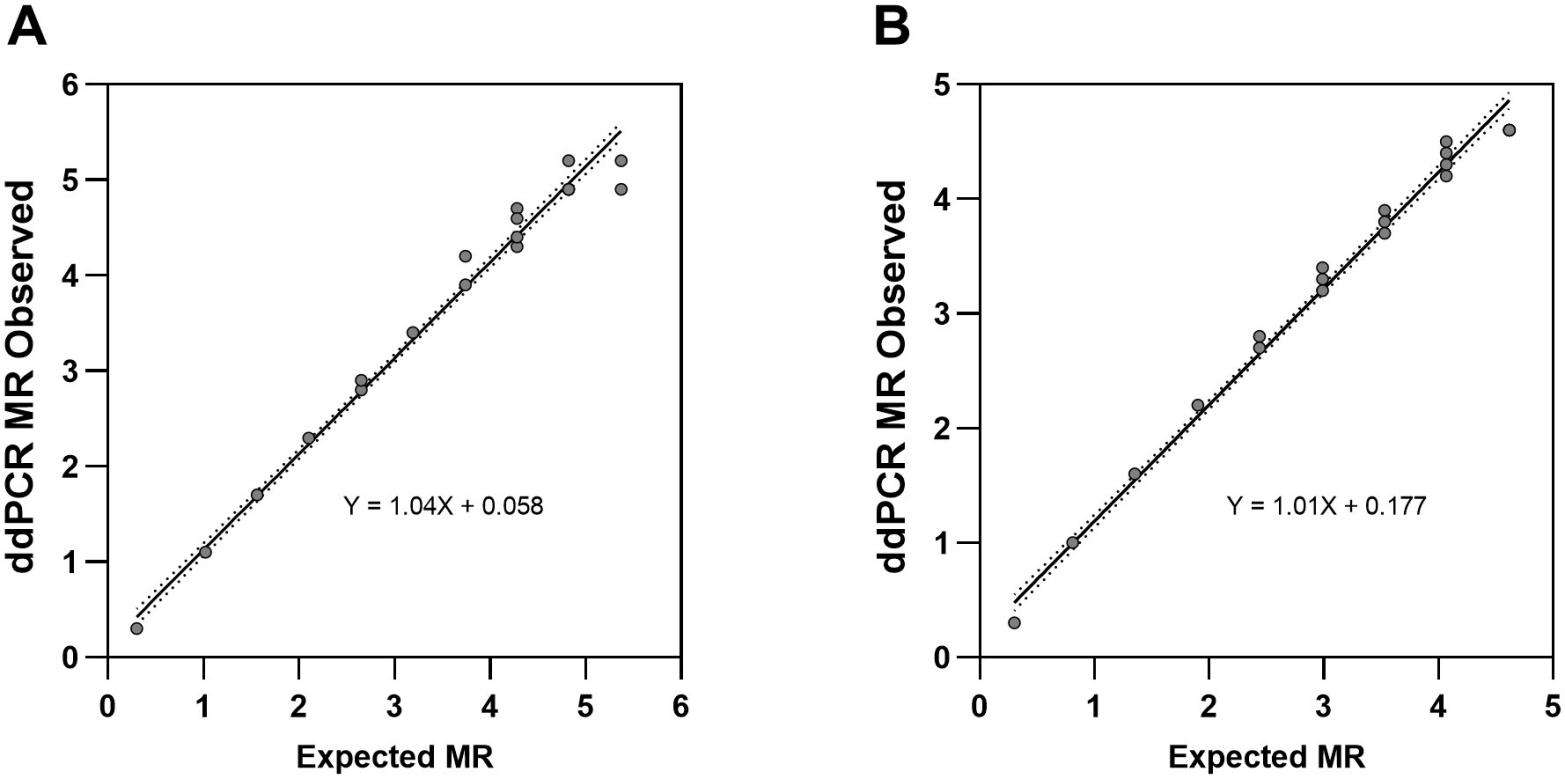
Linearity by breakpoint. A. The e13a2 transcript was diluted from MR0.3-MR4.7 with 4 replicates at each concentration shown as grey circles. The solid black line is the linear regression and has a y intercept equal to 0.1218, slope of 1.004 and a R^2^ of 0.99. B. The e14a2 transcript was diluted from MR0.3-MR4.7 with 4 replicates at each concentration shown as grey circles. The solid black line is the linear regression and has a y intercept equal to 0.1730, slope of 1.015 and a R^2^ of 0.9919.

ddPCR is linear throughout the measuring range of 50%-0.002% in %IS ratio, and MR0.3-MR4.7 in log-space (Fig 3). Variant e13a2 had a measured range of MR0.3 to MR5.32 with a maximum SD of 0.21. Variant e14a2 had a measured range of MR0.3 to MR4.73 with a maximum SD of 0.13. Additionally, 2^nd^ and 3^rd^-order polynomial regression fits were assessed. Deviation from first order linearity was within acceptable limits [S5 Table]. Linearity of MR result was demonstrated from at least MR 0.3 (50%IS) to MR 4.7 (0.002%IS). The e13a2 variant had a slope of 1.04, y-intercept of 0.058 and a R^2^ = 0.996 (p< 0.0001). The e14a2 variant demonstrated similar performance with a slope of 1.01, y-intercept of 0.177 and a R^2^ = 0.993 (p< 0.0001).

### Traceability to WHO-IS

**Fig 4:**
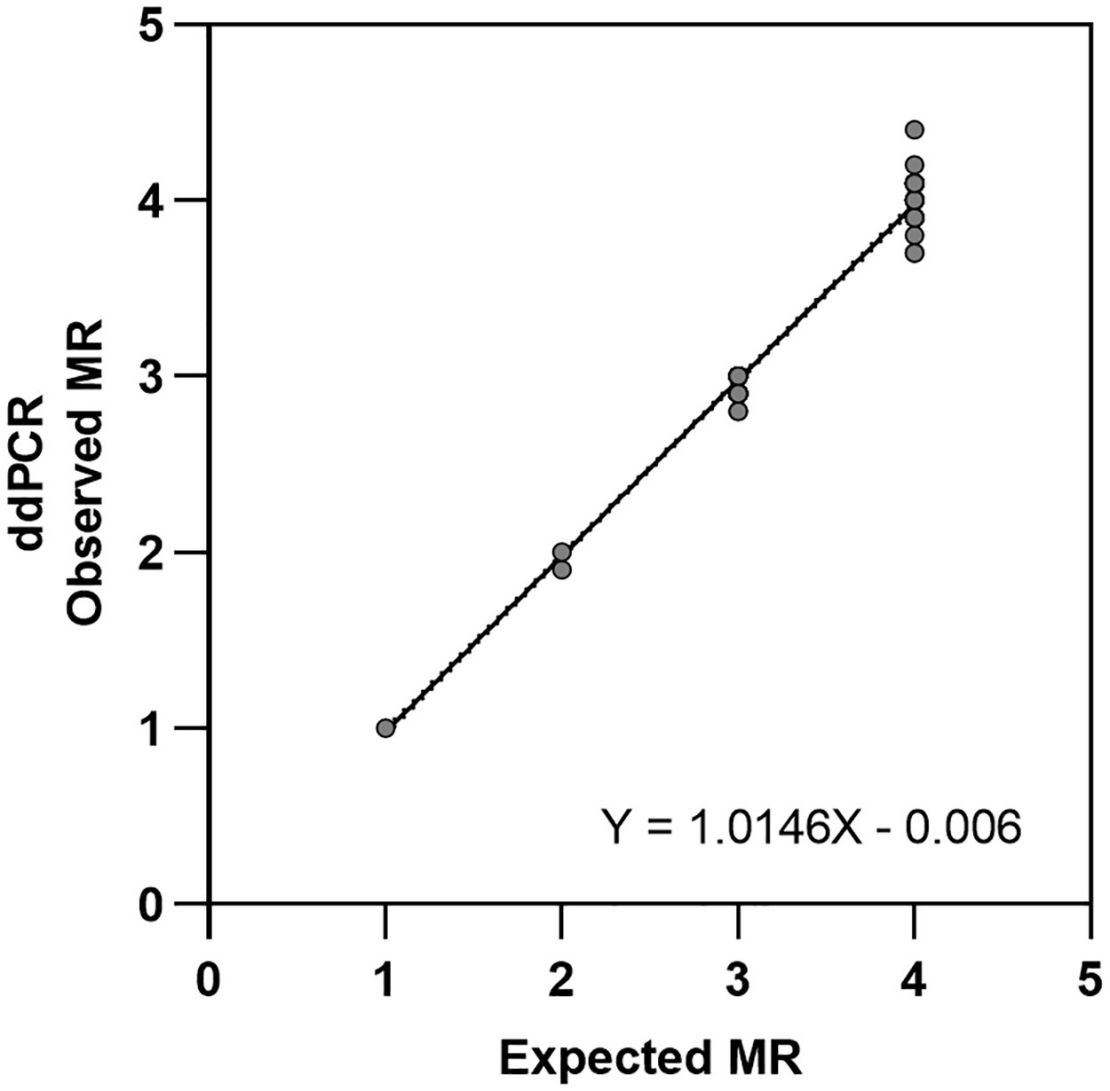
WHO-IS standards. Combination of the individual data from all seven-assay kit lots (solid grey circles) followed by linear regression (solid black line) demonstrated an overall strong correlation (R^2^ = 0.995) with a slope equal to 1.0146 and intercept of -0.00579.

The measured MR values for each level of the WHO Reference Panel were adjusted by a common derived correction factor, CF = 0.93 and compared to the published MR values through a regression analysis to determine slope and intercept values. The analysis showed excellent correlation with R^2^ values of 0.992–0.999. The slope of the regression lines varied between 0.978 and 1.04, and the intercepts were between -0.07 and 0.059. In all cases, the WHO standards measured within the allowable range of their actual assigned values from WHO. Combination of the individual data from all seven-assay kit lots followed by regression analysis demonstrated an overall strong correlation (R^2^ = 0.995) with a slope equal to 1.0146 and intercept of -0.00579 (p< 0.0001) (Fig 4).

### RNA isolation method comparison

Mean MR values, SD and % CV for each dilution and for each RNA extraction method, Maxwell, TRIzol and QIAmp are shown in Table 3A, and an ANOVA analysis comparing the 3 extraction methods is shown in Table 3B. The MR2, MR3 and MR4 samples with the 3 extractions methods showed no significant difference. A three-way ANOVA across the 3 samples and the 3 extraction methods was performed where a non-significant p-value was obtained for all comparisons, shown in Table 3B.

**Table 3:** A. RNA isolation method comparison. B. RNA isolation method comparison ANOVA.

**A. RNA Isolation Method Comparison**				
**Test Type**	**Extraction Method**	**n**	**Mean MR**	**SD MR**
**Reference**	Maxwell	2	2.26	0
**Test**	TRIzol	2	2.3	0.05
**Test**	QIAmp	2	2.28	0
**Reference**	Maxwell	3	3.32	0.12
**Test**	TRIzol	3	3.35	0.06
**Test**	QIAmp	3	3.33	0.07
**Reference**	Maxwell	3	4.08	0.14
**Test**	TRIzol	3	4.39	0.44
**Test**	QIAmp	3	4.37	0.34
**B. RNA Isolation Method Comparison ANOVA**				
**Extraction Method**	**Difference in MR Value**	**Lower confidence Interval**	**Upper Confidence Interval**	**p-value**
**TRIzol- Maxwell**	-0.02	-0.24	0.21	0.98
**QIAmp-Maxwell**	0.06	-0.16	0.27	0.78
**QIAmp—TRIzol**	0.07	-0.16	0.31	0.70

### Multi-site reproducibility

The reproducibility for MR, including repeatability, within-day precision, within-site precision was estimated for all reproducibility panel members. The %CVs for all samples was less than 5% with a maximum observed SD of 0.21 MR (4.85%CV). (Table 4). There was no clear dominant contributor to variation, with the similar SD returned from the individual components including repeatability, within-day, and within-site. The precision of the ddPCR on the run controls and %IS calibrator checks was also evaluated [S6 Table]. The %CV for %IS varied from 3.5% for the sample with the highest *BCR*::*ABL1*%IS (H-CTRL) to 15% for the sample with the lowest *BCR*::*ABL1*%IS (L-CTRL).

**Table 4:** Multi-site clinical reproducibility (N = 36).

Sample	Variant	Target MR	Observed MR (mean)	Repeatability SD	Within-Day SD	Within-Site SD	Reproducibility SD
**C01**	e13a2	0.7	0.7	0	0	0	0
**S07**	e13a2	1	0.98	0.037	0.044	0.044	0.044
**C03**	e14a2	1	1.03	0.037	0.046	0.046	0.047
**S01**	blended	1.2	1.35	0.037	0.043	0.043	0.061
**S08**	blended	2	1.99	0.022	0.022	0.022	0.024
**S02**	e13a2	2.4	2.42	0.037	0.047	0.047	0.047
**S09**	e13a2	2.5	2.5	0.017	0.017	0.017	0.017
**S03**	blended	2.7	2.78	0.033	0.037	0.043	0.043
**S10**	e14a2	3.1	3.14	0.047	0.055	0.055	0.062
**S04**	blended	3.2	3.29	0.064	0.064	0.064	0.064
**C04**	e13a2	3.7	3.44	0.074	0.074	0.074	0.074
**S11**	e14a2	3.5	3.54	0.062	0.073	0.073	0.073
**S05**	blended	3.4	3.62	0.091	0.093	0.093	0.093
**C02**	e14a2	3.7	3.7	0.084	0.084	0.092	0.098
**S06**	blended	4	4.16	0.195	0.195	0.195	0.195
**S12**	e13a2	4.2	4.23	0.183	0.197	0.197	0.205

### Clinical method comparison

**Fig 5:**
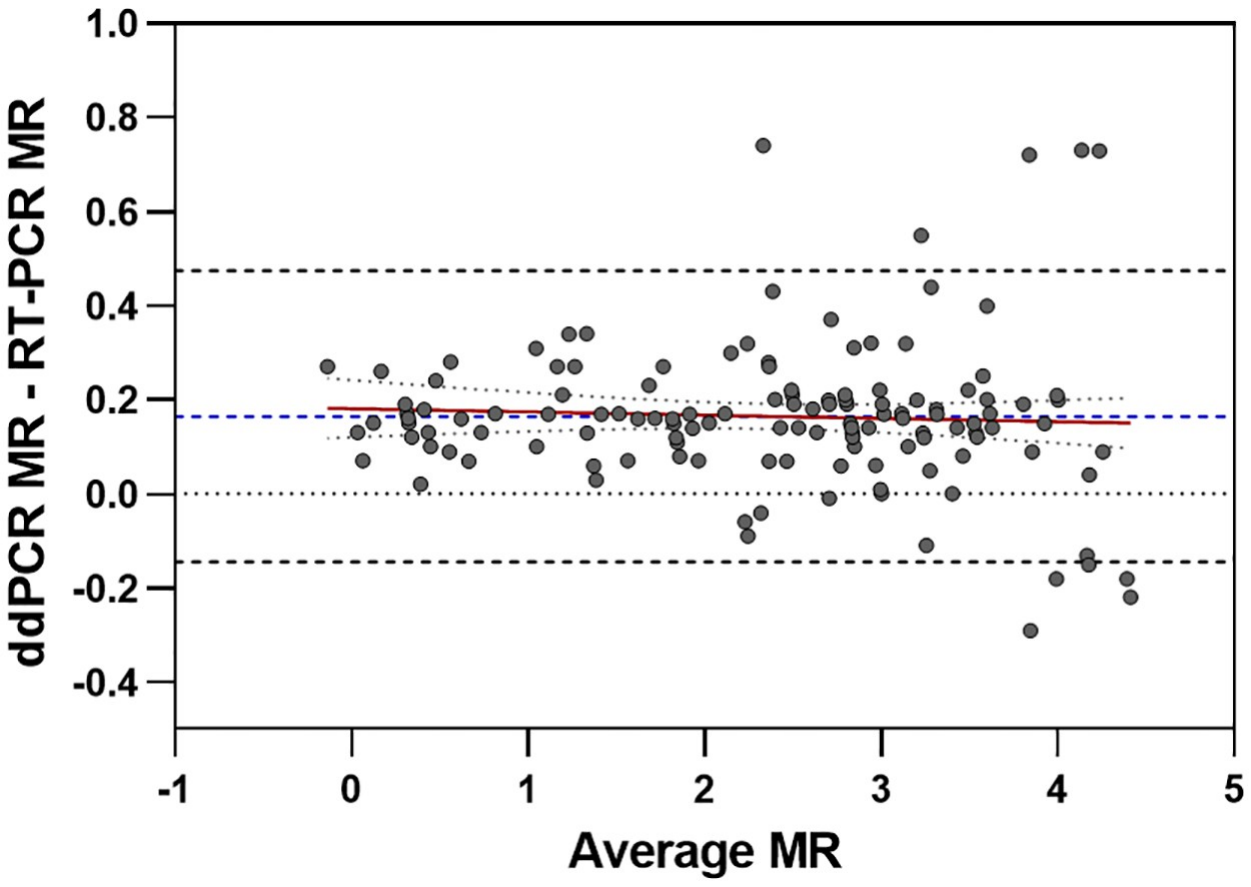
Bland-Altman plot of the 139 evaluable paired samples. The mean bias (95%CI) is shown as a dashed blue line, the limits of agreement are represented by dashed grey lines. The slope of the regression line is indicated by a solid red line.

A comparative evaluation of the accuracy and concordance between ddPCR and RT-PCR on their ability to quantitate *BCR*::*ABL1*/*ABL1* levels was performed using 155 clinical samples, with *BCR*::*ABL1*/*ABL1* levels ranging from not detected to greater than >1MR in the RT-PCR assay per CLSI EP09-A3. Bland Altman and Weighted Deming regression analysis were used to examine systematic differences in variance between the two methods and anticipated differing variances per MR level. Weighted Deming regression requires that all observations are positive; therefore, these analyses excluded pairs in which one or both measurements were non-positive. Of the 155 samples, non-evaluable results were obtained for 16 samples. Three were excluded due to replicate samples, 2 were negative by both technologies, 3 were negative by ddPCR but positive by qPCR, 4 were below LOD or LOQ in one or the other technology and 4 were excluded due to sample quality fails [S7 Table]. For the 139 evaluable paired samples, the mean bias using a Bland-Altman was 0.16 ddPCR- qPCR (95%CI: 0.14–0.19, p<0.0001) (dashed blue line, Fig 5). The limits of agreement (0.47 to -0.14) represent the interval that is expected to contain 95% of the data from an approximately normal distribution and are represented by dashed grey lines. The slope of the regression line, indicated by a solid red line, was -0.007 with a 95% CI of -0.030, 0.016 (p = 0.5533, not significant) and the intercept was 0.1811 (95%CI: 0.120–0.242, p<0.0001).

**Fig 6:**
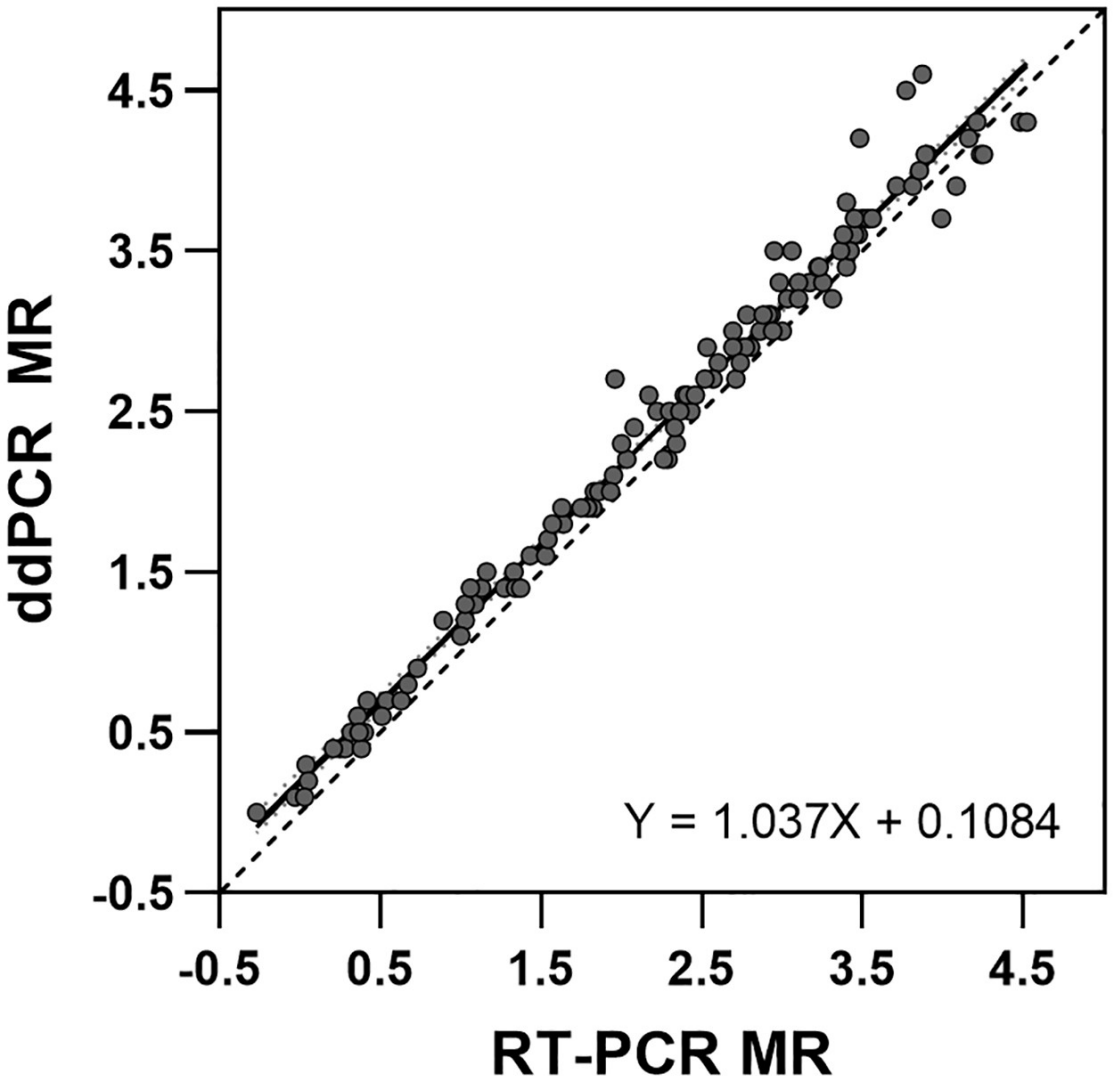
Deming regression analysis of clinical method comparison. Correlation between ddPCR and RT-PCR on 139 evaluable paired samples (solid grey circles). The linear fit is the solid black line, and the identity is the dashed black line.

The ddPCR assay showed excellent linear correlation with the predicate assay using a weighted Deming regression with a Pearson R correlation coefficient of 0.9908, slope 1.037 and intercept was 0.1084 (p = 0.06 for slope and p = 0.32 for intercept) (Fig 6). Separate analyses examined differences between e13a2 and e14a2 variant samples and saw no significant difference between variant types for reporting out across the assay range (data not shown) [S8 Table].

**Fig 7:**
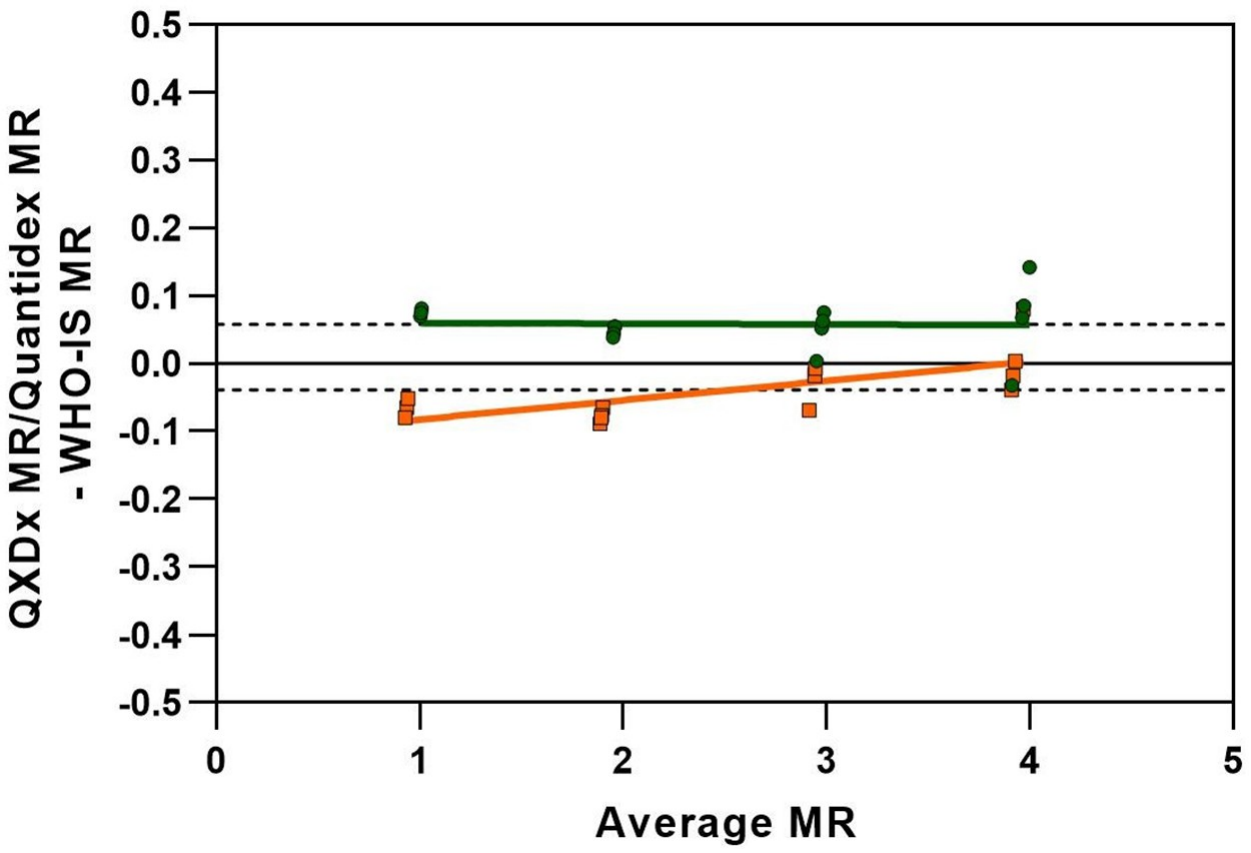
WHO-IS method comparison. Correlation between ddPCR and qPCR on WHO-IS Samples. The ddPCR linear fit is the solid green line and the qPCR linear fit is the solid orange line, the mean bias for each method is shown in the dotted black line which were 0.058 for ddPCR and -0.039 for qPCR.

To further examine the bias difference seen between the predicate method and ddPCR, WHO-IS samples at the 4 MR levels (MR1, 2, 3, 4) were included and run on both systems in replicates of 4. As shown in Fig 7, the RT-PCR (orange line) and ddPCR (green line) methods yielded results on opposite sides of the WHO-IS expected results, accounting for the small bias of 0.16 from predicate (Fig 7). Despite these small differences, both methods yielded a strong and positive relationship between the expected WHO-IS values by fixed effects analysis (p < 0.0001).

## Discussion

We describe a ddPCR assay with excellent sensitivity, specificity, precision, and accuracy, with similar linearity for both *BCR*::*ABL1* fusion transcripts found in the vast majority of CML cases. The assay does not detect the minor or mu fusion transcripts, which represent <1% of CML cases. The assay is aligned to and reports out on the WHO International Standard, with a CF of near 1. In this study performance between sites and operators showed low variability in ddPCR, a challenge for many qPCR-based assays, yet essential for long term monitoring. A study used 10 *BCR*::*ABL1* positive samples across 11 labs, showed higher inter-lab CV% in RTq-PCR when compared head-to-head with RTddPCR [35]. The sum of these performance characteristics led to FDA regulatory clearance.

Sample quality and upstream handling play a large role in the quality of monitoring results, and as such, should be validated carefully by the end user. This includes the critical RNA extraction step. We validated 3 commonly used methods; column-based, bead-based, and organics-based extraction methods. We saw no significant difference in methods across the assay range, with an expected increase in variance of each method at deeper MR samples due to low and single molecule subsampling variability. For specificity we examined several possible sources of false positives: reagents, assay, and biology. All studies yielded highly specific and reproducible results. In the study examining specificity in thirty-six CML negative samples across 2 lots, 3 replicate tests yielded 1 positive droplet each from the same patient. This is likely due to normal biology, consistent with several studies examining low rates of *BCR*::*ABL1* in healthy individuals that were most likely a sporadic generation of the translocation. The presence of these rare transcripts in non-leukemic populations increases in frequency with age [36–38].

In the method comparison with the FDA-cleared qPCR test, 155 samples were tested across the assay range. Sixteen (16) were excluded (3 protocol deviations, 2 were negative by both technologies, 3 negatives by ddPCR but positive by qPCR, 4 were below LOD or LOQ for either of the technologies, and 4 due to sample quality fails) leaving 139 evaluable paired results. For the negative and discrepant samples, the *BCR*::*ABL1* copies being detected are in the single copy range, and therefore this level of subsampling variation is both expected and reasonable. ddPCR demonstrated an overall bias of 0.16 MR in this study with these lots. The calibration to the IS occurs on a lot-by-lot basis for both kits and some slight variation between manufacturing processes is normal. Further investigation of both methods against WHO-IS primary samples indicated slight biases from the WHO on opposite sides of truth, fully accounting for the 0.16 bias observed. Both NCCN and ELN have conservative guidelines on how *BCR*::*ABL1* level trends influence treatment decisions. The biological variation of the assay is considered 0.5 log per test, with 2 successive tests in a trend of >1 log recommended before changing treatment [26]. This general guidance is even more nuanced depending on the individual patient situation. For example, any level of positivity, even at the limit of detection, would be an infinite increase over a prior undetectable test, but would not affect clinical management, whereas a smaller change in a patient with a higher *BCR*::*ABL1* level and a higher clinical risk of relapse (e.g., a high Sokal score), might be more clinically concerning. So, the bias detected in this comparison is well within normal biological and clinical variation and should not affect the treatment decision tree.

There are several fundamental characteristics of ddPCR which may translate into its excellent technical performance. These include, 1) measuring actual molecules (positive droplets) rather than estimating starting template amount by amplification slope and standard curve samples; 2) insensitivity to sample inhibition due to the use of endpoint counting and droplet chemistry rather than amplification slope, the latter easily influenced by poor quality template and/or endogenous and exogenous interferents that might inhibit amplification, and 3) sampling a large number of droplets, which increases the precision of the assay and can increase the sensitivity of the assay. These characteristics also add robustness to the assay performance and allow high levels of reproducible results lab to lab, operator to operator, lot to lot, and even assay to assay [22, 35, 39–43].

The measurement of disease burden via *BCR*::*ABL1* transcript levels has three major clinical implications. First, the decline of disease after initiation of TKI therapy is related to good long-term response. Thus, patients who have *BCR*::*ABL1* above 10%IS after three months of treatment (i.e., a poor early molecular response) have a worse long-term response compared to cases with a *BCR*::*ABL1* of less than 10%IS. Secondly, those patients who achieve a *BCR*::*ABL1* level of <0.1%IS (a major molecular response, MR3) enjoy a “safe harbor” where relapse or progression is quite rare. Lastly, patients who achieve a stable deep molecular response (MR4 or below) can potentially discontinue TKI therapy. Several trials have demonstrated that roughly 50%-80% of such cases can remain without therapies for years [17, 44, 45]. However, this discontinuation strategy relies on carefully and frequently monitoring the patient for disease recurrence, so that the TKI can be reinitiated before the disease bulk arises significantly, as such patients might be at higher risk of progression if early intervention is not started. Prognostic factors for entering TFR studies are still being evaluated and debated. Durable DMR is considered one of the most critical prognostic factors for durable response to going off TKI therapies, in addition to duration of TKI therapy. Accurate measurement of DMR appears to lead to increased prognostic indications for successful cessation; one study has shown that absolute copies of *BCR*::*ABL1* were detected in MR^4^, MR^4.5^, and MR^5 “^undetectable” qPCR cases, which led to better segregation of durable DMR classes, and a statistically significant increase in the probability to predict TFR [17]. Multivariate analysis indicated that digital PCR was the only parameter associated with TFR, though duration of DMR >5 years approached statistical significance. Droplet digital PCR was shown to be a powerful predictor of the ability to predict successful TFR in the U.S. LAST study, a large study of 174 CML patients in DMR who underwent subsequent discontinuation [18]. Patients had conventional RT-PCR performed as the study standard, but also had ddPCR done prior to discontinuation. Of the patients with undetectable *BCR*::*ABL1* by RT-qPCR, 56 (39%) had *BCR*::*ABL1* detected by ddPCR. Patients who were negative by both methods had only a 10% risk of subsequent relapse after discontinuation, while patients who were negative by RT-PCR, but positive by ddPCR, had a 60% relapse rate. Further studies examining DMR and TFR in a clinical setting are ongoing.

The ability to accurately monitor disease burden during therapy, commonly referred to as minimal/measurable residual disease (MRD), is fundamentally important in CML as well as other leukemias (ALL, AML, MPN, APL) and transplant monitoring [46–50]. Additionally, MRD monitoring by liquid biopsy is actively being investigated for solid tumors (NSCLC, CRC, Breast, Melanoma) [51–55]. Indeed academic, regulatory, and pharmaceutical interests are aligning to use accurate and sensitive disease monitoring as early measures of drug efficacy. The use of ddPCR should play an important role in these efforts.

## Supporting information

S1 Table Cross-reactivity with p190 and p230. (DOCX)

S2 Table LOQ analysis. (DOCX)

S3 Table Single-site precision source variability. (DOCX)

S4 Table Accuracy of ERM control material. (DOCX)

S5 Table Linearity. (DOCX)

S6 Table Multi-site precision of calibrator checks and controls. (DOCX)

S7 Table Non-evaluable samples in method comparison. (DOCX)

S8 Table E13 and E14 variants in deming regression fit vs reference method asuragen RT-qPCR. (DOCX)
