# Supplementary material for: Performance characteristics of the first Food and Drug Administration (FDA)-cleared digital droplet PCR (ddPCR) assay for BCR::ABL1 monitoring in chronic myelogenous leukemia
Source: PLoS One. 2022 Mar 17;17(3):e0265278. doi: 10.1371/journal.pone.0265278 (PMC8929598; doi:10.1371/journal.pone.0265278)
Supplement: S1 Table — (DOCX) [file pone.0265278.s001.docx]

**S1 Table. Cross-Reactivity with p190 and p230**

| **Variant Sample ID** | **Dilution Number** | **N** | **Specific Assay Ratio%** | | **QXDx BCR-ABL %IS** | | **% Specificity** | |
| --- | --- | --- | --- | --- | --- | --- | --- | --- |
|  |  |  | **Mean** | **% CV** | **Mean** | **% CV** |  |  |
| p190 | 1 | 4 | 36.1329% | 2.1% | 0.000% | 0.000% | | 100.000% |
|  | 2 | 4 | 0.7148% | 5.3% | 0.000% | 0.000% | | 100.000% |
|  | 3 | 4 | 0.0749% | 10.7% | 0.000% | 0.000% | | 100.000% |
|  | 4 | 8 | 0.0080% | 42.8% | 0.000% | 0.000% | | 100.000% |
| p230 | 1 | 4 | 31.7429% | 1.1% | 0.000% | 0.000% | | 100.000% |
|  | 2 | 4 | 0.6207% | 2.5% | 0.000% | 0.000% | | 100.000% |
|  | 3 | 4 | 0.0630% | 11.0% | 0.000% | 0.000% | | 100.000% |
|  | 4 | 8 | 0.0056% | 63.4% | 0.000% | 0.000% | | 100.000% |
