# Supplementary material for: Performance characteristics of the first Food and Drug Administration (FDA)-cleared digital droplet PCR (ddPCR) assay for BCR::ABL1 monitoring in chronic myelogenous leukemia
Source: PLoS One. 2022 Mar 17;17(3):e0265278. doi: 10.1371/journal.pone.0265278 (PMC8929598; doi:10.1371/journal.pone.0265278)
Supplement: S2 Table — (DOCX) [file pone.0265278.s002.docx]

**S2 Table. LOQ Analysis**

| **Sample Type*** | **Lot** | **Variant** | **Target IS Ratio** | **MR** | **IS %CV** |
| --- | --- | --- | --- | --- | --- |
| Pool 3 - Dilution 0 (neat) | B | e14a2 | 0.01350 | 3.87 | 16.94 |
| Pool 1 - Dilution 0 (neat) | B | Blended | 0.01320 | 3.88 | 27.43 |
| Pool 1 - Dilution 0 (neat) | C | Blended | 0.01200 | 3.92 | 23.68 |
| Pool 2 - Dilution 0 (neat) | C | e13a2 | 0.01180 | 3.93 | 24.55 |
| Pool 1 - Dilution 0 (neat) | H | Blended | 0.01100 | 3.93 | 24.58 |
| Pool 2 - Dilution 0 (neat) | B | e13a2 | 0.01160 | 3.93 | 25.93 |
| Pool 3 - Dilution 0 (neat) | I | e14a2 | 0.01150 | 3.94 | 13.91 |
| Pool 2 - Dilution 0 (neat) | I | e13a2 | 0.01140 | 3.94 | 20.18 |
| Pool 1 - Dilution 0 (neat) | I | Blended | 0.01110 | 3.95 | 24.32 |
| Pool 2 - Dilution 0 (neat) | H | e13a2 | 0.01030 | 3.99 | 26.21 |
| Pool 3 - Dilution 0 (neat) | H | e14a2 | 0.01010 | 4.00 | 33.00 |
| Pool 3 - Dilution 0 (neat) | C | e14a2 | 0.00906 | 4.04 | 35.44 |
| Pool 3 - Dilution 1 | B | e14a2 | 0.00677 | 4.17 | 36.67 |
| Pool 1 - Dilution 1 | B | Blended | 0.00659 | 4.18 | 44.07 |
| Pool 1 - Dilution 1 | C | Blended | 0.00598 | 4.22 | 45.00 |
| Pool 2 - Dilution 1 | C | e13a2 | 0.00590 | 4.23 | 43.40 |
| Pool 1 - Dilution 1 | H | Blended | 0.00587 | 4.23 | 36.36 |
| Pool 2 - Dilution 1 | B | e13a2 | 0.00581 | 4.24 | 43.10 |
| Pool 3 - Dilution 1 | I | e14a2 | 0.00575 | 4.24 | 29.51 |
| Pool 2 - Dilution 1 | I | e13a2 | 0.00572 | 4.24 | 33.33 |
| Pool 1 - Dilution 1 | I | Blended | 0.00555 | 4.26 | 28.33 |
| Pool 2 - Dilution 1 | H | e13a2 | 0.00517 | 4.29 | 42.37 |
| Pool 3 - Dilution 1 | H | e14a2 | 0.00502 | 4.30 | 29.63 |
| Pool 3 - Dilution 1 | C | e14a2 | 0.00453 | 4.34 | 54.05 |
| Pool 3 - Dilution 2 | B | e14a2 | 0.00338 | 4.47 | 65.71 |
| Pool 1 - Dilution 2 | B | Blended | 0.00330 | 4.48 | 60.00 |
| Pool 1 - Dilution 2 | C | Blended | 0.00299 | 4.52 | 50.00 |
| Pool 2 - Dilution 2 | C | e13a2 | 0.00295 | 4.53 | 57.69 |
| Pool 1 - Dilution 2 | H | Blended | 0.00293 | 4.53 | 50.00 |
| Pool 2 - Dilution 2 | B | e13a2 | 0.00291 | 4.54 | 43.75 |
| Pool 3 - Dilution 2 | I | e14a2 | 0.00288 | 4.54 | 42.86 |
| Pool 2 - Dilution 2 | I | e13a2 | 0.00286 | 4.54 | 40.00 |
| Pool 1 - Dilution 2† | I | Blended | 0.00278 | 4.56 | 43.33 |
| Pool 2 - Dilution 2 | H | e13a2 | 0.00258 | 4.59 | 78.26 |
| Pool 3 - Dilution 2 | H | e14a2 | 0.00251 | 4.60 | 39.29 |
| Pool 3 - Dilution 2 | C | e14a2 | 0.00227 | 4.64 | 56.52 |
| Pool 3 - Dilution 3 | B | e14a2 | 0.00169 | 4.77 | 76.47 |
| Pool 1 - Dilution 3 | B | Blended | 0.00165 | 4.78 | 83.33 |
| Pool 1 - Dilution 3 | C | Blended | 0.00150 | 4.83 | 53.85 |
| Pool 2 - Dilution 3 | C | e13a2 | 0.00148 | 4.83 | 60.00 |
| Pool 1 - Dilution 3 | H | Blended | 0.00147 | 4.83 | 66.67 |
| Pool 2 - Dilution 3 | B | e13a2 | 0.00145 | 4.84 | 80.00 |
| Pool 3 - Dilution 3 | I | e14a2 | 0.00144 | 4.84 | 44.44 |
| Pool 2 - Dilution 3 | I | e13a2 | 0.00143 | 4.85 | 57.14 |
| Pool 1 - Dilution 3 | I | Blended | 0.00139 | 4.86 | 75.00 |
| Pool 2 - Dilution 3 | H | e13a2 | 0.00129 | 4.89 | 61.54 |
| Pool 3 - Dilution 3 | H | e14a2 | 0.00126 | 4.90 | 100.00 |
| Pool 3 - Dilution 3 | C | e14a2 | 0.00113 | 4.95 | 58.33 |

***Dilutions 4-7 led to extremely low values for which %CV calculations were not reliable. †Pool 1- Dilution 2 is the relevant data point for determination of LLOQ.**
