# Supplementary material for: Performance characteristics of the first Food and Drug Administration (FDA)-cleared digital droplet PCR (ddPCR) assay for BCR::ABL1 monitoring in chronic myelogenous leukemia
Source: PLoS One. 2022 Mar 17;17(3):e0265278. doi: 10.1371/journal.pone.0265278 (PMC8929598; doi:10.1371/journal.pone.0265278)
Supplement: S3 Table — (DOCX) [file pone.0265278.s003.docx]

**S3 Table. Single-site Precision Source Variability**

| **Target MR** | **Mean MR** | **Within-Run** | | **Between Operator** | | **Between Days** | | **Between Lots** | | **Between Instruments** | | ***N*** |
| --- | --- | --- | --- | --- | --- | --- | --- | --- | --- | --- | --- | --- |
|  |  | **SD** | **% CV** | **SD** | **% CV** | **SD** | **% CV** | **SD** | **% CV** | **SD** | **% CV** |  |
| 1 | 1.40 | 0.022 | 1.60 | 0.013 | 0.94 | 0.010 | 0.70 | 0.017 | 1.20 | 0.007 | 0.53 | 100 |
| 2 | 2.47 | 0.038 | 1.56 | 0.006 | 0.23 | 0.011 | 0.45 | 0.027 | 1.11 | 0.004 | 0.19 | 100 |
| 2.5 | 2.80 | 0.046 | 1.65 | 0.008 | 0.30 | 0.003 | 0.01 | 0.013 | 0.46 | 0.000 | 0.00 | 100 |
| 3 | 3.31 | 0.080 | 2.42 | 0.000 | 0.00 | 0.000 | 0.00 | 0.000 | 0.00 | 0.000 | 0.00 | 100 |
| 3.5 | 3.63 | 0.103 | 2.83 | 0.000 | 0.00 | 0.000 | 0.00 | 0.000 | 0.00 | 0.000 | 0.00 | 100 |
| 4 | 4.13 | 0.162 | 3.94 | 0.011 | 0.29 | 0.000 | 0.00 | 0.000 | 0.00 | 0.012 | 0.28 | 99 |
| 4.7 | 4.65 | 0.242 | 5.22 | 0.000 | 0.00 | 0.000 | 0.00 | 0.042 | 0.90 | 0.000 | 0.00 | 89 |
| <1.0 | 0.73 | 0.068 | 0.93 | 0.000 | 0.00 | 0.000 | 0.00 | 0.018 | 2.4 | 0.057 | 0.78 | 100 |
