# Supplementary material for: Performance characteristics of the first Food and Drug Administration (FDA)-cleared digital droplet PCR (ddPCR) assay for BCR::ABL1 monitoring in chronic myelogenous leukemia
Source: PLoS One. 2022 Mar 17;17(3):e0265278. doi: 10.1371/journal.pone.0265278 (PMC8929598; doi:10.1371/journal.pone.0265278)
Supplement: S4 Table — (DOCX) [file pone.0265278.s004.docx]

**S4 Table. Accuracy of ERM Control Material**

| **Lot** | **Day** | **Copies Expected(log10)** | **QXDx Mean Copies (log10)** | **% CV** | ***N*** |
| --- | --- | --- | --- | --- | --- |
| 1 | 1 | 3.01 | 2.98 | 0.27 | 8 |
| 1 | 1 | 4.01 | 3.99 | 0.17 | 8 |
| 1 | 1 | 2.02 | 1.99 | 1.57 | 8 |
| 1 | 1 | 5.03 | 5.04 | 0.22 | 8 |
| 1 | 1 | 1.00 | 0.99 | 9.74 | 8 |
| 1 | 2 | 3.01 | 3.01 | 0.36 | 8 |
| 1 | 2 | 4.01 | 3.99 | 0.11 | 8 |
| 1 | 2 | 2.02 | 2.00 | 1.23 | 8 |
| 1 | 2 | 5.03 | 5.07 | 0.29 | 8 |
| 1 | 2 | 1.00 | 0.97 | 10.58 | 8 |
| 1 | 3 | 3.01 | 3.02 | 0.50 | 8 |
| 1 | 3 | 4.01 | 4.04 | 0.11 | 8 |
| 1 | 3 | 2.02 | 2.03 | 1.01 | 8 |
| 1 | 3 | 5.03 | 5.07 | 0.34 | 8 |
| 1 | 3 | 1.00 | 1.04 | 9.98 | 8 |
| 2 | 1 | 3.01 | 2.97 | 0.23 | 8 |
| 2 | 1 | 4.01 | 3.98 | 0.18 | 8 |
| 2 | 1 | 2.02 | 2.03 | 0.96 | 8 |
| 2 | 1 | 5.03 | 5.04 | 0.19 | 8 |
| 2 | 1 | 1.00 | 0.97 | 11.72 | 8 |
| 2 | 2 | 3.01 | 3.00 | 0.30 | 8 |
| 2 | 2 | 4.01 | 3.98 | 0.15 | 8 |
| 2 | 2 | 2.02 | 2.01 | 1.44 | 8 |
| 2 | 2 | 5.03 | 5.05 | 0.15 | 8 |
| 2 | 2 | 1.00 | 1.04 | 8.26 | 8 |
| 2 | 3 | 3.01 | 3.02 | 0.15 | 8 |
| 2 | 3 | 4.01 | 4.06 | 0.08 | 8 |
| 2 | 3 | 2.02 | 2.01 | 0.73 | 8 |
| 2 | 3 | 5.03 | 5.07 | 0.29 | 8 |
| 2 | 3 | 1.00 | 1.00 | 8.40 | 8 |
| 3 | 1 | 3.01 | 2.99 | 0.56 | 8 |
| 3 | 1 | 4.01 | 4.01 | 0.09 | 8 |
| 3 | 1 | 2.02 | 2.09 | 1.10 | 8 |
| 3 | 1 | 5.03 | 5.06 | 0.19 | 8 |
| 3 | 1 | 1.00 | 0.99 | 12.20 | 8 |
| 3 | 2 | 3.01 | 2.99 | 0.66 | 8 |
| 3 | 2 | 4.01 | 4.01 | 0.19 | 8 |
| 3 | 2 | 2.02 | 2.01 | 0.97 | 8 |
| 3 | 2 | 5.03 | 5.03 | 0.12 | 8 |
| 3 | 2 | 1.00 | 1.00 | 8.06 | 8 |
| 3 | 3 | 3.01 | 3.00 | 0.39 | 8 |
| 3 | 3 | 4.01 | 4.02 | 0.09 | 8 |
| 3 | 3 | 2.02 | 2.06 | 0.61 | 8 |
| 3 | 3 | 5.03 | 5.03 | 0.29 | 8 |
| 3 | 3 | 1.00 | 0.96 | 8.47 | 8 |
