# Supplementary material for: Performance characteristics of the first Food and Drug Administration (FDA)-cleared digital droplet PCR (ddPCR) assay for BCR::ABL1 monitoring in chronic myelogenous leukemia
Source: PLoS One. 2022 Mar 17;17(3):e0265278. doi: 10.1371/journal.pone.0265278 (PMC8929598; doi:10.1371/journal.pone.0265278)
Supplement: S5 Table — (DOCX) [file pone.0265278.s005.docx]

**S5 Table. E13 and E14 Variants in Linear vs Nonlinear Model Comparison**

| **Variant** | **Sample ID** | **Mean Expected MR** | **Mean Measured MR** | **Predicted MR 1^st^ Order** | **Predicted MR 2^nd^ Order** | **Predicted MR 3^rd^ Order** |
| --- | --- | --- | --- | --- | --- | --- |
| E13 | Positive Pool – Dilution 0 | 0.30 | 0.30 | 0.37 | 0.30 | 0.30 |
|  | Positive Pool – Dilution 1 | 1.10 | 1.02 | 1.12 | 1.11 | 1.10 |
|  | Positive Pool – Dilution 2 | 1.70 | 1.56 | 1.68 | 1.70 | 1.69 |
|  | Positive Pool – Dilution 3 | 2.30 | 2.10 | 2.24 | 2.28 | 2.28 |
|  | Positive Pool – Dilution 4 | 2.83 | 2.65 | 2.81 | 2.86 | 2.86 |
|  | Positive Pool – Dilution 5 | 3.40 | 3.19 | 3.37 | 3.41 | 3.41 |
|  | Positive Pool – Dilution 6 | 3.98 | 3.74 | 3.94 | 3.96 | 3.96 |
|  | Positive Pool – Dilution 7 | 4.50 | 4.28 | 4.50 | 4.48 | 4.48 |
|  | Positive Pool – Dilution 8 | 4.98 | 4.82 | 5.06 | 4.99 | 4.99 |
| E14 | Positive Pool – Dilution 0 | 0.30 | 0.30 | 0.48 | 0.32 | 0.33 |
|  | Positive Pool – Dilution 1 | 1.00 | 0.81 | 1.00 | 0.96 | 0.95 |
|  | Positive Pool – Dilution 2 | 1.60 | 1.35 | 1.55 | 1.59 | 1.58 |
|  | Positive Pool – Dilution 3 | 2.20 | 1.90 | 2.10 | 2.20 | 2.20 |
|  | Positive Pool – Dilution 4 | 2.73 | 2.44 | 2.65 | 2.77 | 2.77 |
|  | Positive Pool – Dilution 5 | 3.28 | 2.99 | 3.21 | 3.30 | 3.31 |
|  | Positive Pool – Dilution 6 | 3.80 | 3.53 | 3.76 | 3.79 | 3.80 |
|  | Positive Pool – Dilution 7 | 4.35 | 4.07 | 4.31 | 4.25 | 4.25 |
|  | Positive Pool – Dilution 8 | 4.60 | 4.62 | 4.86 | 4.68 | 4.66 |
