# Supplementary material for: Performance characteristics of the first Food and Drug Administration (FDA)-cleared digital droplet PCR (ddPCR) assay for BCR::ABL1 monitoring in chronic myelogenous leukemia
Source: PLoS One. 2022 Mar 17;17(3):e0265278. doi: 10.1371/journal.pone.0265278 (PMC8929598; doi:10.1371/journal.pone.0265278)
Supplement: S6 Table — (DOCX) [file pone.0265278.s006.docx]

**S6 Table. Multi-Site Precision of Calibrator Checks and Controls**

| **Sample ID** | **Target MR** | **MR (Observed)** | | **Target Level (% IS)** | **% IS (Observed)** | | | ***N*** |
| --- | --- | --- | --- | --- | --- | --- | --- | --- |
|  |  | **Mean** | **SD** |  | **Mean** | **SD** | **% CV** |  |
| H-CTRL | 1.0 | 0.70 | 0.0000 | 20 | 19.1925 | 0.6773 | 3.5 | 40 |
| ~10% IS | 1.0 | 1.13 | 0.0460 | 10 | 7.2887 | 0.3801 | 5.2 | 38 |
| ~0.1% IS | 3.0 | 3.09 | 0.0578 | 0.1 | 0.0827 | 0.0087 | 10.6 | 38 |
| L-CTRL | 3.5 | 3.50 | 0.0733 | 0.03 | 0.0321 | 0.0048 | 15.0 | 40 |
