# Supplementary material for: Performance characteristics of the first Food and Drug Administration (FDA)-cleared digital droplet PCR (ddPCR) assay for BCR::ABL1 monitoring in chronic myelogenous leukemia
Source: PLoS One. 2022 Mar 17;17(3):e0265278. doi: 10.1371/journal.pone.0265278 (PMC8929598; doi:10.1371/journal.pone.0265278)
Supplement: S7 Table — (DOCX) [file pone.0265278.s007.docx]

**S7 Table. Non-evaluable Samples in Method Comparison**

| **Sample** | **Bio-Rad Result** | **Asuragen result** | | | **Exclusion Reason** |
| --- | --- | --- | --- | --- | --- |
| 7 | BCR-ABL not detected | | Detected | Low BCR-ABL copy Range | |
| 10 | Fail ABL insufficient | | Below LoD | Low ABL, differential ABL cut-off | |
| 12 | BCR-ABL not detected | | Detected | Low BCR-ABL copy Range | |
| 19 | BCR-ABL not detected | | BCR-ABL not detected | Low BCR-ABL copy Range | |
| 22 | BCR-ABL not detected | | Below LoD | Low BCR-ABL copy Range | |
| 24 | BCR-ABL not detected | | BCR-ABL not detected | Low BCR-ABL copy Range | |
| 25 | Detected | | Below LoD | Low BCR-ABL copy Range | |
| 45 | Detected | | Detected | Duplicate Sample | |
| 48 | Detected | | Detected | Duplicate Sample | |
| 51 | Detected | | Detected | Duplicate Sample | |
| 90 | BCR-ABL not detected | | Detected | Low BCR-ABL copy Range | |
| 98 | Fail ABL insufficient | | Detected | Low ABL, differential ABL cut-off | |
| 107 | BCR-ABL not detected | | Below LoD | Low BCR-ABL copy Range | |
| 118 | Fail ABL insufficient | | Fail no ABL 1 | Low ABL, differential ABL cut-off | |
| 127 | Error | | Positive (above ULOQ) | Poor Phenotype | |
| 145 | BCR-ABL detected below LLOQ | | Detected | Low BCR-ABL copy Range | |
