# Supplementary material for: Performance characteristics of the first Food and Drug Administration (FDA)-cleared digital droplet PCR (ddPCR) assay for BCR::ABL1 monitoring in chronic myelogenous leukemia
Source: PLoS One. 2022 Mar 17;17(3):e0265278. doi: 10.1371/journal.pone.0265278 (PMC8929598; doi:10.1371/journal.pone.0265278)
Supplement: S8 Table — (DOCX) [file pone.0265278.s008.docx]

**S8 Table. E13 and E14 Variants in Deming Regression Fit vs Reference Method Asuragen RT-qPCR**

| **E13** | **N** | **Estimate** | **Lower 95% CI** | **Upper 95% CI** |
| --- | --- | --- | --- | --- |
| **Intercept** | 139 | 0.15495 | 0.06238748 | 0.2292047 |
| **Slope** |  | 1.007257 | 0.96886832 | 1.0590709 |

| **E14** | **N** | **Estimate** | **Lower 95% CI** | **Upper 95% CI** |
| --- | --- | --- | --- | --- |
| **Intercept** | 139 | 0.1915187 | 0.1463140 | 0.2451814 |
| **Slope** |  | 0.9835591 | 0.9567462 | 1.0027981 |
